# Australian chiropractic sports medicine: half way there or living on a prayer?

**DOI:** 10.1186/1746-1340-15-14

**Published:** 2007-09-19

**Authors:** Henry Pollard, Wayne Hoskins, Andrew McHardy, Rod Bonello, Peter Garbutt, Mike Swain, George Dragasevic, Mario Pribicevic, Andrew Vitiello

**Affiliations:** 1 Macquarie Injury Management Group, Department of Health and Chiropractic, Macquarie University, NSW 2109, Australia

## Abstract

Sports chiropractic within Australia has a chequered historical background of unorthodox individualistic displays of egocentric treatment approaches that emphasise specific technique preference and individual prowess rather than standardised evidence based management. This situation has changed in recent years with the acceptance of many within sports chiropractic to operate under an evidence informed banner and to embrace a research culture. Despite recent developments within the sports chiropractic movement, the profession is still plagued by a minority of practitioners continuing to espouse certain marginal and outlandish technique systems that beleaguer the mainstream core of sports chiropractic as a cohesive and homogeneous group. Modern chiropractic management is frequently multimodal in nature and incorporates components of passive and active care. Such management typically incorporates spinal and peripheral manipulation, mobilisation, soft tissue techniques, rehabilitation and therapeutic exercises. Externally, sports chiropractic has faced hurdles too, with a lack of recognition and acceptance by organized and orthodox sports medical groups. Whilst some arguments against the inclusion of chiropractic may be legitimate due to its historical baggage, much of the argument appears to be anti-competitive, insecure and driven by a closed-shop mentality.sequently, chiropractic as a profession still remains a pariah to the organised sports medicine world. Add to this an uncertain continuing education system, a lack of protection for the title 'sports chiropractor', a lack of a recognized specialist status and a lack of support from traditional chiropractic, the challenges for the growth and acceptance of the sports chiropractor are considerable. This article outlines the historical and current challenges, both internal and external, faced by sports chiropractic within Australia and proposes positive changes that will assist in recognition and inclusion of sports chiropractic in both chiropractic and multi-disciplinary sports medicine alike.

## Background

Orthodox medicine in Western nations has evolved essentially into a two-tiered structure with general practitioners and specialists. In addition, a number of ancillary health professional groups have arisen to service the ongoing co-management needs of individuals under care. In this way physiotherapists and others have contributed to the health care system in a supportive role. In parallel to this development, a number of complementary and alternative health care professions have arisen in separation from orthodox medicine, amongst which chiropractic has become the most established [1-3]. In similar fashion to orthodox medicine, chiropractic has developed its own 'specialty' groups, including sports chiropractic [4].

The past 20 years has seen an explosion in the sophistication of sports medicine and sports science within Australia. An awful performance by Australia at the 1976 Montreal Olympic Games where Australia failed to win a gold medal (regarded as a severe national embarrassment by the Government of the day) saw the establishment of the government funded Australian Institute of Sport (AIS) in 1981. Whilst sports medicine was born in the United States of America (USA), its development in Australia set the standard for the world, with the demand for and recognition of Australian sports medicine and sports science growing to an unprecedented high [5,6]. In addition, professional sport within Australia continues to grow in popularity and professionalism [7], reflected by the growing budgets for team medical and fitness services and equipment [8].

Over the last 20 years, sports infrastructure has developed to a degree that professions involved in the management of sports health (medicine, physiotherapy, nutrition, podiatry, sports sciences and psychology) have all evolved subspecialty groups. This development has occurred at a pace that has outpaced local chiropractic evolution. It is likely that this is one reason for the difficulty that chiropractic has encountered in being included and recognized in organized sports medicine. Confounding this development has been the individual approach by some high profile chiropractors making some extravagant claims of therapeutic effect through the application of individual management approaches.

This paper will discuss what sports chiropractic is, how it differs to standard chiropractic and outline both the historical and current internal and external challenges faced by sports chiropractic. Positive changes will be proposed, that will hopefully assist in the recognition and inclusion of sports chiropractic in chiropractic and multi-disciplinary sports medicine alike.

## What is sports chiropractic – how does it differ to general chiropractic?

Whilst not true of the entire profession, traditional general practitioner chiropractors (GPCs) have been primarily concerned with the osseous components of patient's complaints [9,10], while sports chiropractors have given more consideration to both the hard and soft/connective tissues (muscle, tendon, ligament, fascia etc) [11]. It should be noted that while the majority of GPCs have incorporated both passive and active forms of therapy in patient management, a small proportion of GPCs use a "classical" approach of uni-modal therapy interventions [12,13] (namely manipulation only, and often in one single style) in the management of spinal conditions. It is the opinion of the authors that this polarised management approach along with the often vocal and politically active "classical" GPCs fearing a lack of unified professional identity, that has contributed to a "spine only" or spine specialist role being proposed as the model for the chiropractic profession [14].

### Sports chiropractic is not manipulation

Sports chiropractors are often considered to be uni-modal practitioners with limited regard for orthodox medical approaches [15]. However, it appears from the literature that chiropractors are not limited to this uni-modal, manipulation only approach, as patient management appears to be predominantly multi-modal, particularly in the field of sports chiropractic [11-13]. This "modern" multi-modal (MMM) chiropractic management has been said to incorporate components of passive and active care to address both the acute inflammatory/pain phase and the chronic/rehabilitation/injury prevention phase [11]. Hoskins et al. [11] have stated that such management typically incorporates a combined approach of various manual therapy procedures with an emphasis on high velocity low amplitude techniques, massage and stretching techniques, rehabilitation and therapeutic exercises (including proprioception exercises, motor pattern correction and sport specific rehabilitation), and non-local biomechanical improvement (including orthotic intervention) to improve the kinematic and kinetic chain function. Other modalities used include taping, physical therapies (such as ice and heat modalities), electrotherapeutics, acupuncture, gait retraining, nutrition, footwear/ergonomic/training advice and exercise/cross training programs. Thus, the MMM approach of sports chiropractors is condition and patient specific, and goes beyond the symptomatic improvement of local tissues to addresses non-local factors that may be important in injury aetiology or injury recurrence [16].

Despite the majority of chiropractors actually utilising a multi-modal approach [12,13], the minority of uni-modal practitioners is often thought as being typical of the profession [17]. This limitation is often cited as a reason for the exclusion of chiropractic from organizations such as Sports Medicine Australia (SMA) [17]. It appears that much of this concern has more to do with the politics of exclusion than it has to do with the minority of practitioners whom operate in that fashion.

A potential solution for this impasse could be to establish a code of practice for sports chiropractors consistent with published sports chiropractic viewpoints [18]. By agreeing to attain certain minimum standards of care and application, sports chiropractors would fulfil all the requirements of inclusion into organizations such as SMA whilst protecting the athletic and sports medicine communities at the same time. Although some chiropractors may see such restrictions as unreasonable, a negotiated stand between all the groups would ensure an adequate outcome for all. Clearly, attempts for the past 15–20 years by the sports chiropractors to attain full member status in SMA have failed. This lack of success is highlighted by the successful recent inclusion of osteopathy into the SMA organization. A success said to be due in part to the perception that osteopaths are "team players", are more biomechanical in their approach and are safer in the application of their techniques (personal communication). A change of approach by chiropractic is required after a period of introspection and evaluation.

### Sports chiropractic approach

The sports chiropractor acknowledges and has assimilated a large body of clinical information unique to the diagnosis and management of the special needs of those who participate in sport. This includes being highly familiar with the vast array of sports injuries which may be incurred by an athlete in their particular sport. They use this information in delivering treatment which, in many ways, does not resemble the traditional care rendered by GPCs. Consultations tend to be longer and are characterised by active care management strategies focused on the specific needs of the injury under consideration. In many ways sports chiropractic approaches resemble ancillary medical approaches. The typical approach of the sports chiropractor is to perform a diagnostic triage to rule out red flag conditions, diagnose and treat symptomatic tissues and recognise and evaluate functional deficiencies and aetiological factors responsible for factors causing sports injury [11]. They use traditional orthopaedic and neurological testing procedures to inform their investigation as well as more traditional chiropractic assessments that include: structural analysis, palpation (motion and static) and range of motion testing along with referral for radiological analysis or advanced imaging and other specialist services if required. Moreover, the sports chiropractor acknowledges limitations and contra-indications to care and has a strong understanding of the referral basis for advanced imaging or special testing and actively partakes in inter-professional communication and co-operation [19-30]. In addition, many sports chiropractors now participate successfully in post surgical rehabilitation and management programs for spinal and peripheral joint procedures [26,27,31-36].

### The dualistic nature of chiropractic management

Some classical GPCs de-emphasise pain management in favour of "wellness" care [37]. However, the treatment of the acute inflammatory/pain phase of injury is an important consideration in the management of injury by sports chiropractors. Some GPCs emphasise both factors, whilst others tend to emphasise pain/inflammation management. As such, there is a dualistic nature in the management of sports injury by chiropractors. What is important is the fact that one approach cannot come at the expense of the other. Both are required. Given the nature of sporting injuries and their onset, pain and inflammation is the usual presentation, causing athletes to request of the practitioner to "give me something for the pain". This presentation must be managed appropriately before performance can be considered. It is a hierarchical approach to injury management [38,39].

Although the sports chiropractor can perform the basic functions of a GPC, a requirement exists for sports chiropractors to distinguish themselves as a specialist group. This distinction must come through detailed knowledge of sports, sports specific injuries, their mechanisms and management, and the issues surrounding athletic performance enhancement. This knowledge and understanding of athletes and sport should exceed that of the GPC. It is no longer good enough to claim sports specialist status without an increased level of understanding. However, this statement should not preclude the GPC from treating sports injuries any more than what occurs with the general practice physiotherapist as undergraduate programs teach examination, diagnosis and treatment of sports injuries. The implementation of standardised post graduate training in sports specific injury management includes knowledge of the sporting rules, sports injury epidemiology, injury mechanism and an understanding of the psyche of the athlete amongst other sports specialist knowledge areas [40]. Central to the concept of the specialist is an expert knowledge of the soft tissues and the appendicular structures. A thorough understanding of the anatomy, biomechanics, motor patterns, and kinetic chains is required [16]. Such an orientation would be expected of any practitioner claiming to be able to manage sports injuries today. In medical practice, where a general practitioner may attempt to manage a sports person, athletes are frequently referred to other practitioners for expert advice or treatment. In the same way, whilst any chiropractor may be able to render a diagnosis and treatment to an athlete, additional special skills and abilities unique to the sports chiropractor should surpass those of the GPC.

### Philosophy

It is likely that the sports chiropractor has a differing practice philosophy than the GPC. It has been stated as a definition for the GPC that chiropractic is "the science of locating offending spinal structures, the art of reducing their impact to the nervous system, and a philosophy of natural health care based on your inborn potential to be healthy" [10,41]. However, a more appropriate definition of sports chiropractic is that it focuses upon the acquisition of maximised athletic performance and superior injury management through the application of the highest quality of chiropractic management, treatment, rehabilitation and prevention of sports related injury [42]. This is achieved through education, training and clinical research into the cause, treatment, rehabilitation and prevention of sports related injuries and the enhancement of athletic performance [42]. These goals are similar to those expressed by other professions [43]. The sports chiropractic definition would seem to incorporate the evolving role of evidence based practice and how it must be implemented into sports chiropractic practice. The GPC is more likely to embrace the historically traditional philosophy of chiropractic, whereas the sports chiropractor is likely to place a far greater emphasis on pain management, soft tissue management and exercise rehabilitation [42], with less focus on dogmatic and dated philosophies espoused by some classical GPCs [44,45].

Of particular note for the chiropractic profession is the pursuit of the wellness paradigm in the sports practice. Whilst it is the contention of the authors that the primary role of the sports chiropractor is to provide management of pain and inflammation and promote the return of the injured athlete back to sport, it is also the goal to maximise performance [46]. It is likely that the chiropractic profession should more easily embrace the wellness model in a sporting context and recognise it as the equivalent of the concept of the "promotion of performance" in the sporting arena. This is important as many classical GPCs speak of sports chiropractors in demeaning terms because they choose to treat pain and inflammation rather than focus on a non-pain based wellness paradigm. This is a bewildering position given that rates of chiropractic use among athletes is higher per capita than in non-athletes and this evidence supports the view that such pain management is both common and important to chiropractors [47]. In recognising the role of performance, the schism between the classical GPC and the sports chiropractor could be minimised. The promotion of peak performance in athletes (with appropriate measures of outcome [46]) is a form of wellness care and its pursuit is consistent with the historical philosophy of chiropractic [44].

## Education

Modern chiropractic educational programs producing GPCs are at least 5 years (double degree bachelor [48-50] or bachelors/masters programs) of full time tertiary study covering all the areas of study typically taught in physiotherapy programs (minus the surgical and hospital based components not amenable to the chiropractic scope of practice e.g. stroke rehabilitation, cardiopulmonary physiotherapy etc.). These should be recognised for the fact that they produce graduates with of good competency in musculoskeletal examination, diagnosis and management. However, the philosophy they embrace when they leave university renders it their choice which side of the dualistic nature of chiropractic they wish to embrace.

There are political sensitivities with sports chiropractic education due to the resultant competition created between educational providers, particularly those between local university programs and international programs (non Australian post professional qualifications). The competition extends to the political infrastructure associated with them.

The advancement of sports chiropractic on the world stage is important and should be controlled by one administrative organisation. That organisation is the Federation of International Chiropractic Sportive (FICS). The selection to international sporting events should meet minimum standards as approved by one overarching organization such as FICS. Qualification for such participation is currently proposed to be the International Diploma in Chiropractic Sport Science (ICSSD) [51]. Conflict has occurred in the past when chiropractors with university based sports degrees that supersede the ICSSD or the preceding program; certified chiropractic sports practitioner (CCSP), were not considered for appointment. However, this scenario has changed in recent years with such individuals being able to acquire the ICSSD qualification through the granting of credit transfer as is typical in most university programs. The role of FICS at the international level is not in dispute. However, the role of FICS at the national level must be one that is carefully considerate of local variables of education, political development (of chiropractors and other competitive groups such as physiotherapists and osteopaths), funding and acceptance by the public. Whilst the ICSSD may be appropriate for some countries as the minimum standard, it may not be in others. It is likely for a variety of reasons that the ICSSD should not be the minimum standard of entry for the specialty of sports chiropractic in Australia.

In Australia FICS is not yet recognized by the sports medicine community[52]. Unfortunately, this is also true of the national sports chiropractic group and chiropractors in general[52]. An ongoing concern that has been expressed by many chiropractors (Hyde, 2006, personal communication) is that the objectives of the international organization do not adequately address the concerns of the local practitioners in terms of scope of practice or educational level. It is imperative that adequate national standards are met by local practitioners, otherwise progression to national representation in teams under the control of the national sports medicine organization (SMA) will not be accepted and hence, chiropractors will not be selected for duties in state or national teams. Until this occurs, such practitioners will not be selected for national duty and therefore would never come under the jurisdiction of the international organization FICS. It is the opinion of the authors that in order to progress the international standing of Australian chiropractors, all efforts should be made to align with the local organising body SMA. By doing so, chiropractors would be more likely to be selected for duty in international competitions. Inherent in this national involvement is the requirement of sports chiropractors to maintain standards commensurate with those of their peers in sports medicine and sports physiotherapy.

### Future education

Many could view the lack of inclusion of chiropractors into organizations such as SMA as anticompetitive behaviour as the basic professional education of chiropractors is the equivalent of other qualifying professions [53]. So whilst the discipline of sports chiropractic should evolve into a post graduate specialty, inclusion into SMA should not be predicated on this as it is not a requirement of other professions.

However, in the context of acquiring a specialisation the appropriate educational program would be based in a university, supported by the local professional association(s), and offered at graduate diploma level at the least. This approach is based on the educational system of the jurisdiction. In Australia, all education in health care occurs in the Government based university system. Expectations in Australia are that education is provided at a university level and offerings that are not university based are held in lower regard. Currently, the chiropractic profession in Australia uses the ICSSD (a private international program of study) as the sports related base qualification. This situation recently changed with the commencement of a post graduate diploma sports chiropractic offered by Murdoch University. This program followed masters level programs in sports chiropractic from Macquarie and RMIT universities.

In keeping with movements in other professions, the educational level to be adopted for a sports specialisation status should be elevated above the offering of a post graduate certificate program to the level of a graduate diploma or greater. In time, true specialty status could be achieved with elevation to a masters qualification with a research component. Mootz [54] has suggested that specialist competency should have residency as a part of its training. In his editorial he has stated that mature residency and fellowship opportunities are urgently needed in virtually any area outside of chiropractic's perceived core nonsurgical spine care competencies if fields such as sports chiropractic are to ever achieve sustainable credibility. Such training is preferred and is being applied in a sports context in the 1000 hour Canadian Sports Fellowship Program (SFP) from Canadian Memorial College Chiropractic (CMCC) [55]. That such a model could be adopted internationally and qualifications issued via a recognised consortium of Universities would address all issues of standardised care delivery, content, level and type of qualification could be addressed.

Unlike previous offerings, the key to the success of such a program in attracting candidates lies in the ability of the convenors to imbed significant relevant practical content into the theoretical offerings. In order to achieve this integration, it is likely that the associations and special interest groups (Sports Council, FICS etc) should integrate some of their activities into the progressive training of the specialists. Such activity follows the lead of other specialist training programs in traditional disciplines as well as newer disciplines such as the sports physicians and sport physiotherapists [7,56].

With recognition of sports chiropractic as a specialty group, a requirement exists for funding dollars to be set aside to sports chiropractic research projects [11]. In particular, the creation of injury surveillance and other clinical data to support the existence of sports chiropractic, and its relatively safe and effective nature should be expanded from the small base that it now occupies. The lesson of the homogenous delivery of care needs to be learnt with the cessation of the unsubstantiated claims of brilliance from individuals pushing their particular technique barrow.

### A tiered system of practitioners and funding for service

An elevation of the sports chiropractic education program above that of the traditional training, leads to the potential for creating a tiered system of practitioners. This system better reflects the specialisations occurring in other health disciplines of medicine and physiotherapy. In such a system, there exists the intern (a possible category for those educational programs offering a preceptorship), the general practitioner and the specialist. With a clear delineation between these groups being achieved through education and practical experience, the public could readily understand the differences between the groups as well as support a tiered payment system. Such a system if established could encourage the establishment of candidates in the educational programs as well as demonstrate to the public the growing and expanding and mainstream nature of the profession. Implicit in this categorisation must be support by the educational and political groups associated with the profession.

The minimum entry level standard for specialisation status should be elevated to the level of a masters program after an establishment period of time, as is typical of other professions. By working together, professional associations and continuing education bodies may form the bridge between the initial graduate diploma and the masters degree.

### Barriers to the establishment of specialisation programs

Considerable barriers exist to the uptake of sports chiropractic in this country. In order for the sports programs to gain their due recognition outside of the chiropractic profession, the programs must overcome these barriers by becoming university based, masters level, evidence based, research driven and contain challenging sports specific material in a practical environment.

A challenge in establishing such programs will be staffing them with appropriately qualified academics [57]. Whilst many educators are available for the task, few educators exist that have post-graduate qualifications from a university in a sports related discipline and have the practical credibility to gain traction with potential candidates in the proposed program. It is likely (and desirable) that multi-disciplinary specialist assistance will be required in the delivery of these programs if they are to be accepted by the sports medicine community. This real and potentially costly issue will face educational institutions. The added benefit of this assistance will be the integration of professionals who are already members of the governing sports bodies. This integration may facilitate better communication, understanding and implementation of sports chiropractic to the wider sports medicine organization.

The lack of speciality status during the pursuit of specialist educational training will have a significant direct and opportunity cost to participants in time and lost earnings. Factors such as these may reduce the likelihood of participation from the practitioner level, impacting on numbers in the proposed programs and their ultimate viability. Apart from the self satisfaction gained from attaining a degree and improved education, there is currently no professional status or financial incentive to attract participants.

As programs are presented by different educational providers, a potential exists for variability in the scope and level of the programs. It will be important that consensus is achieved on what an entry level specialist training program should contain, much like those that exist for the training of entry level chiropractors in Australia [58,59].

Moreover, once practitioners have achieved post graduate sports chiropractic education and even the specialisation status, there is a lack of a recognised higher fee for service for specialists rendering treatment as specialist sports chiropractors (as opposed to traditional chiropractors treating the odd sports injury without the requisite training). Insurance and other third party payors would need to recognize the training associated with specialization and reward them with greater reimbursement. However, such recognition is predicated on political activity from associations and registration boards as well as academic involvement.

However, the acknowledgement and acceptance of sports specialists and their high quality training programs have been slow to say the least in other fields of sports medicine by other professional bodies such as medical schools, the Royal Australian College of General Practitioners, the Australian Medical Council and the Health Insurance Commission [7,60]. To state that sports chiropractic will face hurdles in its lofty goal of specialization status is an understatement to say the least: more likely a bed of nails than a bed of roses, for the foreseeable future.

Furthermore, there is no protection of the title 'sports chiropractor'. Current legislation does not prevent the general chiropractor from titling his or her practice 'sports chiropractic' usually as a function of a company or business name. Subsequently due to this lack of protection in title, the consumer may be falsely drawn to a GPC with inferior qualifications and knowledge in an arena directly relevant to sports chiropractic. Inappropriate or substandard care rendered by such practitioners may potentially be associated with genuine sports chiropractic and have a negative effect via athlete dissatisfaction. Whilst this is a matter for concern, this limitation is true of other professions as well.

The profession has a duty to document its treatment efficacy in the form of scientific evidence in the literature. This has begun [11], but the published peer reviewed literature lags behind other professions but probably exceeds some. Despite this start of a base of literature, a requirement exists for practitioners wishing to be involved in the immediate future to contribute to the scientific research and literature. Difficulty though it may be, a contribution from all will help address a generation of inactivity, bridge the gap that has been created by the activity of other orthodox sports medicine groups and help contribute to a positive research driven culture for the future of sports chiropractic.

## Historical internal problems

It is likely that the causes of problems in sports chiropractic are both internal and external to chiropractic. Mistakes; we have made a few. It is fair and reasonable to suggest that perhaps chiropractic has traditionally not played well with the other members of the sports team (Mitchell, 2006, personal communication). The chiropractic profession has a long history of individual approaches to recognized sports medicine organizations and a lack of well thought out, structured and professionally orientated approach (CAA sports interest group meeting Sydney December 2005, Personal communication).

An unfortunate history exists of individuals going down in a blaze of glory with their own "unique" behaviours emphasising specific techniques rather than evidence based approaches. Not only has this damaged the profession by presenting a splintered facade to the other members of the sports medicine team, but once there, the behaviour of some of these individuals has demonstrated a lack of ability to relate with other professionals or more seriously, a lack of willingness to do so. A serious concern within the sports medicine community is the strong perception that some chiropractors are unable to work within a team as an equal member and pay due respect to the other professionals within that team for their area of expertise [61]. This concern continues to create barriers to the acceptance of chiropractors within the multidisciplinary sports medicine setting.

### The future of sports chiropractic

Chiropractic now acknowledges that the road to participation must include the TEAM approach. The individual grandstanding and grandiose claims of unreal performance enhancement were made a long time ago. Claims should be supported by evidence. In fact extraordinary claims should be supported by extraordinary evidence. The only true currency in modern health care is evidence. Those in chiropractic often opine that we have served our sentence but committed no crime. Honest reflection must reveal a truth that we (or individuals representing the "we") have espoused such practices in the past. Recognition of such activity and the potential developmental growth associated with such reflection is important. Rejection of unsupported practice through the application of modern practice is essential for future inclusion in the sports medicine organisations.

## Historical external problems

It is a widely accepted belief that successful sports performance is acquired through a multi-disciplinary sports medicine effort [62,63]. However, it has been stated that an environment in sports medicine has been created where a true integrated, multidisciplinary environment is at best difficult to foster or at worst impossible to achieve [62]. This is bad medicine and a result of the different sports medicine professions developing to a point where each discipline is now relatively isolated and separate from the other, with historical conflicts between and within disciplines and an "us versus them" mentality [62]. Considering this, it would be reasonable to expect that sports chiropractic would face some difficulty in becoming accepted by some aspects of the more established orthodox sports medicine team. But to what level should this reasonably be expected and tolerated and for how long?

SMA (previously known as the Australian Sports Medicine Federation) founded in 1963, is the peak national umbrella body for sports medicine and sports science in Australasia. It is widely acknowledged overseas as the world's leading multi-disciplinary sports medicine body. To be eligible for SMA full membership applicants must have completed a three year full time tertiary degree that is recognized and approved by the SMA National Board [52]. The professions of most full members are: physiotherapists, general practitioners, sports doctors, sports physicians, exercise scientists, sports dietitians, sports pscyhologists, podiatrists, physical activity academics and researchers, public health experts, orthopaedic surgeons and physical education teachers. Beneath the level of full membership is a subordinate category known as an associate membership for anyone with an interest in sports medicine, sports science, physical activity or public health. It is at this level that chiropractors with their double degree programs are eligible for membership along with others such as massage therapists, coaches, officials and administrators. By contrast, osteopaths have recently been elevated from associate membership to full membership but chiropractors were not. This is despite the five or six year chiropractic university based, private practice focused education being of longer duration than the majority of professions entitled to full membership. This lack of recognition has seen chiropractors typically not considered for appointments to major sporting competitions such as the Commonwealth and Olympic Games and a large section of professional sport within Australia and the government funded AIS. It is a shame that the true embodiment of the multidisciplinary charter that is the purvey of the AIS excludes the participation of qualified university trained Australian practitioners. This is curious, as the driving force behind the formation of the multidisciplinary AIS was to redress the awful performance by Australians at the Olympics that preceded its Charter. Perhaps there is a lesson in that for all of us.

Chiropractic's exclusion from the Australian sports medicine cognoscenti appears to be the result of behavioural excesses of a few chiropractors in the past. It appears somewhat unreasonable that a whole profession should be excluded rather than simply establish a professional code of conduct to control such behaviour as outlined in other prestigious groups such as the American College of Sports Medicine [64] where chiropractors can become full members.

For over 20 years the chiropractic profession has been involved in a long standing battle to gain recognition with SMA as full members [17]. This lack of status appears to be a problem inherent and unique to Australia as chiropractors face no impediment in applying for full membership in other international sports medicine organizations such as the American College of Sports Medicine, Sports Medicine New Zealand and the South African Sports Medicine Association. Why is it only Australia that has remained steadfast against chiropractic inclusion?

It has been stated that some SMA members and former members see their interests as threatened by the existence of perceived 'rival' groups or by the umbrella of the organization itself [65]. Other authors have discussed the closed shop mentality elsewhere in Australian medicine [66]. It also appears that there has been institutional bias by organised sports medicine groups including the SMA to stop the inclusion and progression of chiropractic into the sports arena [17]. Numerous examples exist of chiropractors losing access to teams and organizations once their professional identity was revealed [17]. Also true are the examples of individuals who were given full membership to SMA because of an undergraduate degree, only to have it rescinded to the lower associate member status once a second degree in chiropractic and a professional qualification in chiropractic was attained [17].

The reasons stated by SMA to exclude chiropractic from full membership have differed with time and were documented by Simpson [17]. In his paper which is still relevant today, Simpson states that the initial reasons for the decision were that the SMA committee found two areas in chiropractic philosophy and principles of which it felt were incompatible with the philosophies of the health professionals who make up the full membership:

• Chiropractors practice a 'healing science' capable of treating nearly the entire range of human ailments [17].

• Chiropractor's practice 'maintenance' and prescribe spinal adjustments on a regular basis largely in pursuit of the above [17].

At the time the chiropractic profession demonstrated that these arguments were flawed. The example of 'maintenance' chiropractic as the basis of exclusion is remarkable and an example of 'the pot calling the kettle black' given that the physiotherapy profession supports and conducts 'maintenance' physiotherapy [67]. In fact the majority of physiotherapy provided at national and international sporting events appears to be of an asymptomatic nature [68]. Intriguing, as stated by Flanagan & Green [67], 'maintenance' physiotherapy has seemingly been based on the chiropractic model [69].

Despite their justification being challenged, SMA remained unwavering on chiropractic's exclusion. Later though, SMA's position changed and they conveyed that their decision was unanimous in its determination to decline the request for full membership to suitably qualified chiropractors for the following reasons:

• They had an unashamed admittance that the major bodies supporting the organization would cease to do so if chiropractors were accepted as full members. They, being the Australian physiotherapy Association (APA) and the Australian Medical Association (AMA), represented not only the significant majority of the scientific and financial contribution but also the majority of participation in the organisational structure. It is likely that this threat of a boycott remains today and is likely to be a large contributing factor to the continued reluctance to allow chiropractic entry into the organization.

• That members of the chiropractic profession still practice unscientific methods. This is a value judgement. It is based on anecdotal evidence of individual effort that is extrapolated to a whole profession. Such extrapolation is flawed and does not sit well in the era of evidence based practice. The comment is surprising given that Orchard & Brukner [7] state that so much of sports medicine is not supported by evidence and it lags somewhat behind some of the more traditional specialties. It appears reasonable that one who fosters a certain level of expertise should actually demonstrate it or suffer the same fate as those that are victims of it.

• Concerns that chiropractors would not provide any service expertise that was not already available from medical and physiotherapy members. This comment is a double edged sword for those that use it as a justification of such policies. The fact that chiropractors are said not to offer anything that is different implies by definition that many of the procedures are the same and therefore should be accepted for being so. Recent evidence has demonstrated that chiropractic is not only different in its clinical application to manipulative physiotherapy [70] but other evidence also exists separating the various manual therapy professions [71]. This concern is also addressed within a recent publication by the World Health Organisation (WHO). In their publication on the status of chiropractic, they not only examined some of what separates chiropractors from other health professionals, but go as far as to make recommendations on what level of study would bring another health practitioner up to the same level of competence as a chiropractor. "For medical doctors and other health care professions, the duration of training depends upon credits from previous education and experience, but not less than 2,200 hours over a two or three-year full-time or part time program, including not less than 1000 hours of supervised clinical training [72]." Recent literature has also contradicted the viewpoint that physiotherapists provide the same service as chiropractors in that mobilization and/or manipulation is rarely performed at sporting events by physiotherapists [68], whereas its use is widespread by sports chiropractors [70]. Whilst many physiotherapists are qualified as manipulative physiotherapists, it appears as if the majority of techniques used by such practitioners are slow velocity in nature [73]. If high velocity techniques are used, according to Jull [74] and others [75] they are done so sparingly, possibly due to a degree of paranoia and hysteria within the profession regarding the dangers of high velocity manipulation [76,77]. In days gone by the chiropractic profession was snubbed in the sports arena and maligned for its use of manipulation and the dangers associated with it. Apparently, physiotherapy doctrine would have us believe that physiotherapy manipulation is safer. A fact not borne by the evidence [78]. Finally, many athletes, including professional athletes [79,80], actually prefer to see chiropractors for some conditions [4,47].

### "Don't mention the war..."

Of the reasons stated for the exclusion of chiropractors is that it is highly likely that the biggest resistance is from the more established physiotherapy profession. The point should be made that it has been cited that the bias is occurring more at the professional level through the APA rather than at the individual practitioner level [17].

Despite this frequently cited potential reaction to chiropractic inclusion, it is unlikely that it would ever eventuate. The only losers in such a display would be the physiotherapists. Saner heads would prevail and both professions would co-exist, unhappily at first. Thereafter, barriers would break down and real cooperation and communication would result in an environment of professional and friendly rivalry. A situation analogous to the competitive environment that exists between the athletes we all treat, an environment that would encourage innovation, cooperation and real progression between the professions for the benefit of all concerned. Now, what's wrong with that? Why not look forward to an era of cooperation rather than persecution as already demonstrated by our professions [81].

Furthermore, it often appears that the bias towards chiropractic is institutionalised. Many a physiotherapist has graduated (and later worked with a chiropractor) and admitted they had no first hand knowledge of what a chiropractor is or does (Fitzgerald, Australian Institute of Sport 1999, Personal communication). Essentially, little is known about what a chiropractor does or what they are trained to do. Furthermore, it is the authors' opinion that the sports medicine community have numerous misconceptions about the average sports chiropractor that is based on individual experience at best or the conduct of classical GPCs. What they do know has evolved through games of "Chinese whispers" that has resulted in second, third and fourth hand information. The same can be said of the chiropractic profession regarding the physiotherapy profession. Ignorance is not something that should be broadcast, by either group. Is it not time for everyone to take a reality check and enter the 21^st ^century? When all is said, there is much more in common with the professions than there are differences [82].

The perceived turf war between chiropractic and physiotherapy has resulted in a change of direction from a model of athlete-centred care to profession-centred care. Most sports chiropractors can recall an example of how they were removed from a team because of intervention by physiotherapists. This has continued for many years and seems not to be abating. Are sports practitioners so insecure of their skills?

Why does this occur? Increasingly, it appears that such decisions are being driven by self-interested anti-competitive issues rather than true athlete-focused issues. Whilst this segregation of chiropractors from athletes is a win for practitioner centred care, it is an unqualified failure for the athlete or patient centred model of practice. It should be noted that without the athletes and sport participation, the requirement for employment of sports professionals would not be necessary. Moorhead [65] cites that Professor Barry Brooks has called for professionals from a multitude of disciplines to work collaboratively to provide the best possible care for their clients. The best results will be seen when all disciplines work in a multidisciplinary team [65].

Inherent in this fiasco is an obligation by all to recognise that the tertiary training of Australian chiropractors is provided by physiotherapists, medics, sport scientists and many of the same groups who insist that chiropractic is unscientific. Curious. Does this imply that they are unscientific by association and the groups to which they belong? There are many examples in the university systems where chiropractors are taught the same information in the same classes often by the same people as physiotherapists and osteopaths [40,49,50]. Thus, chiropractors receive very similar education to that of physiotherapists, albeit for one year longer duration, only to hear that the knowledge gained in the training of the chiropractor somehow metamorphoses into something that does not understand the same basic medical principles taught at the undergraduate level once they acquire the title chiropractor. It is likely that a disinterested party could view these anomalies and creative interpretations as anti-competitive behaviour.

It is often stated that chiropractors use unsubstantiated techniques and should therefore not be a part of a team. This argument may be true of some of the techniques used by chiropractic [44,83,84], but it is also true of the other professions as well, such as the 'classical physiotherapy' approach of massage, electrotherapeutics and exercises [85]. The advent of evidence based medicine has detailed the lack of efficacy for many conventional physiotherapy approaches. For example, most electrotherapies probably have little more than placebo effects [86-90]. Evidence to support electrophysical therapies is lacking, despite their use being well established within physiotherapy practice [90]. Ultrasound is the most widely used therapeutic agent to enhance soft tissue healing [88]. Despite this, meta analyses have found it no more clinically effective than placebo in the treatment of musculoskeletal injury [86,88,89]. Consequently, more contemporary physiotherapy literature has seen the documentation and investigation of the usefulness of various manual therapy approaches into the management of peripheral conditions [91,92]. A notable move away from electrotherapeutics and devices has occurred so that the modern physiotherapist now predominantly works with their hands, just like chiropractors. Notwithstanding the academic push to evidence based practice, it has been said that the physiotherapy profession remains reluctant to change their clinical practice [93]. As is likely typical of all professions, the majority of clinical techniques chosen by physiotherapists still remain directed by their initial training [94]. One could reason then that the similar training that each group receives, often by the same people, again makes the professions more similar than what is often portrayed.

### Sports performance care

Another factor creating a degree of frostiness with the physiotherapy profession and SMA is the uncorroborated belief that chiropractors treat patients 'forever'. This is particularly interesting given evidence exists for all professions (chiropractic, osteopathy and physiotherapy) of over-servicing patients [95]. Traditional chiropractic has a central tenet of promoting wellness through spinal manipulative therapy [44]. Traditional sports science/medicine/physiotherapy has a central tenet to promote performance through various means [43,68]. Whilst chiropractors are frequently castigated for a pursuit of wellness care, we contend that the difference between wellness and performance is more semantic than real.

The belief that high-level athletes and teams should receive preventative and ongoing prehabilitation, massage therapy and exercise protocols (core stability and eccentric muscle training protocols) [96] as part of the standard medical/physiotherapy management is an example of this approach. Is this not a wellness concept for the athlete? The benefits of massage therapy are largely based on observations and experiences that massage can provide benefit, much like that in chiropractic. However, very little scientific data has supported performance benefit, injury treatment, injury prevention or recovery resulting from massage [97] or the outcomes of wellness care provided by chiropractors. However, the lack of evidence for efficacy is not the evidence of lacking efficacy.

### Appointment of providers

Despite the exclusion from SMA and other organized bodies, individual chiropractors have managed to keep the faith and demonstrated success in obtaining representation to participate in high-level sporting events or to assist with high-level teams. Often individual participation has been organised by individual patients of those practitioners with athletes directly requesting inclusion of "their" chiropractor as opposed to the generic sports medicine approach (physiotherapist, masseur and if well funded a medic). Such requests are usually only granted to professional teams and highly funded amateur organizations that generally operate outside of organised sports medicine organizations or, the time of the chiropractor is volunteered for free outside of any official selection process (which does nothing to provide an official "track record" for the use of chiropractors with administrators).

Alternatively, chiropractic representation often comes at the expense of the title chiropractor, with the chiropractor being appointed to teams and being able to participate as an official 'massage therapist' (Hodge, New South Wales Institute of Sport 1999–2000, personal communication). Again, such participation affords the use of the chiropractic services without the recognition of such services by the coordinating sports medicine organisation. The loss of title does nothing in terms of acknowledgement of chiropractic in professional sport. However, to date, chiropractors have not been able to secure official participation in organised Australian sports medicine coverage at major national and international sporting events such as the Commonwealth Games and the Olympics. However, FICS was "officially" recognized as providing sports chiropractic at the 2005 World Games in Germany. This situation remains the chiropractic equivalent to the boulevard of broken dreams.

## External to the future

### Organised sport medicine in Australia

Over at least the last seven years, despite an increase in the number of professionals working in sports medicine, SMA membership has declined [65]. The main factor in this membership decline appears to have been the increasing specialisation within sports medicine [65]. It is interesting that during this period, where chiropractors have not been allowed full membership status, that such a decline could be minimised or stalled with the access to greater potential number of members.

The significance of this fact remains that the strength of an organization resides in its numbers. If a national organization that has a unified voice in creating policy for sport, policy for health and political activities (government and non-government) then the absence of chiropractic, a member group of the affiliation of associations can only serve to further isolate chiropractors from inclusion in the broader scope of sporting activity and representation [63].

We speculate that the inclusion of chiropractic into SMA should be seen as a potential to expand the group with the inclusion of many new members. Members that would be more than happy to abide by a code of conduct so long as their professional identity is recognised.

## Conclusion

Sports chiropractic is a sophisticated emerging sub speciality of chiropractic that urgently deserves recognition by general chiropractic as well as other health care providers and their representative groups. Inherent in such recognition is the self-control and self-determination that should come by the application of a modern code of practice and the embodiment of evidence based practice ideals. With the consideration of the above and the recognition and removal of its historical baggage, the time is right for sports chiropractic to evolve and become accepted for the benefit of all concerned particularly the athlete patient.

## Competing interests

No funding was received in the preparation of this manuscript. The authors have no conflict of interest directly related to the content of the manuscript.

## Authors' contributions

HP and WH conceived the idea of the paper. At a series of meetings HP, WH, AM, RB, PG, MS and MP contributed to writing an initial draft document that reflected the collective thoughts and experiences of the participants. Over a course of further meetings and through email, all authors contributed to the writing and re-writing of this paper. All authors made original contributions to the content of the final manuscript. All of the authors participated in the editing and revisions of the multiple drafts that existed between the initial and final draft. All authors read and approved the final manuscript.
